# Case report: Curing a rare, unstable hemoglobin variant Hb Bristol-Alesha using haploidentical hematopoietic stem cell transplantation

**DOI:** 10.3389/fimmu.2023.1188058

**Published:** 2023-06-30

**Authors:** Qin Zhang, Yujia Huo, Qinggang Sun, Nan Liu, Hongchuan Shi, Minghui Wang, Jinming Xiao, Hanzi Yuan, Xiangfeng Tang

**Affiliations:** ^1^ Department of Hematology, No.971 Hospital of People's Liberation Army Navy, Qingdao, Shandong, China; ^2^ Department of Traditional Chinese Medicine, Qingdao Special Service Sanatorium of People's Liberation Army Navy, Qingdao, Shandong, China; ^3^ Department of Pediatrics, the Sixth Medical Center of People's Liberation Army General Hospital, Beijing, China; ^4^ Department of Pediatrics, the Seventh Medical Center of People's Liberation Army General Hospital, Beijing, China

**Keywords:** unstable hemoglobinopathy, hemolytic anemia, Hb Bristol-Alesha, hematopoietic stem cell transplantation, haploidentical

## Abstract

Unstable hemoglobinopathies are a rare, heterogeneous group of diseases that disrupt the stability of hemoglobin (Hb), leading to chronic hemolysis and anemia. Patients with severe phenotypes often require regular blood transfusions and iron chelation therapy. Although rare, studies have reported that hematopoietic stem cell transplantation (HSCT) seems to be an available curative approach in transfusion-dependent patients with unstable hemoglobinopathies. Here, we describe successful haploidentical HSCT for the treatment of an unstable Hb variant, Hb Bristol-Alesha, in a 6-year-old boy with severe anemia since early childhood. Two years after transplantation, he had a nearly normal hemoglobin level without evidence of hemolysis. DNA analysis showed complete chimerism of the donor cell origin, confirming full engraftment with normal erythropoiesis.

## 1 Introduction

Unstable hemoglobinopathies are a rare, heterogeneous group of hemolytic anemias caused by gene mutations in globin chains, leading to alterations in the solubility and stability of hemoglobin (Hb). Gene mutations result in the substitution or deletion of amino acids in the globin subunits. The general mechanisms that destabilize Hb include the weakening or altering of heme-globin interactions, interference with the secondary or tertiary structure of the Hb subunits, or interference with subunit interactions (1). Structure-altered Hbs have an increased tendency to denature and autoxidize to form methemoglobin, which results in the production of hemichromes. Hemichromes induce the clustering of band 3 at their membrane sites; these globular intracellular aggregates are called Heinz bodies (2, 3). The formation of Heinz bodies decreases membrane pliability and increases membrane permeability. Oxidative damage from free heme iron may further compromise the membrane. As a result, red blood cells (RBCs) containing Heinz bodies show reduced deformability and increased fragility (4, 5). The impairment of deformability causes RBCs to be preferentially trapped in the spleen. The spleen removes damaged cells or just a section of the membrane and Heinz body inclusions. Erythrocyte membrane loss reduces the lifespan of affected cells and eventually leads to their removal from circulation. Some Hbs are so unstable that they are difficult to detect in hemolysates because of their rapid denaturation and degradation. These Hbs are called hyperunstable Hb variants. The clinical presentations of the disease vary, ranging from mild to severe hemolytic anemia, depending on the instability of the Hb variants. Mildly unstable Hbs may not cause clinical symptoms; however, severely affected patients require chronic transfusion therapy from infancy or early childhood. Inheritance is autosomal dominant in most cases; however, *de novo* mutations have also been described (1, 6).

Hb Bristol-Alesha results from a G>A mutation at codon 67 of the β-globin gene, with a single amino acid substitution from normal β67 valine (Val) to β67 methionine (Met) or β67 aspartate (Asp). This substitution introduces a highly charged polar residue into the hydrophobic heme pocket, breaking the nonpolar bond between Val and the heme group. This results in the disruption of the molecular stability of Hb, which finally causes severe hemolytic anemia (7, 8). To date, there are no curative options for unstable Hb variants, except hematopoietic stem cell transplantation (HSCT). Although HSCT has been widely described as a therapeutic strategy for hemoglobinopathies such as thalassemia major (TM) and sickle cell disease (SCD), its application is rarely reported for the treatment of unstable hemoglobinopathies. Herein, we report successful haploidentical HSCT (haplo-HSCT) in a pediatric patient with Hb Bristol-Alesha.

## 2 Case presentation

**Figure 1:**
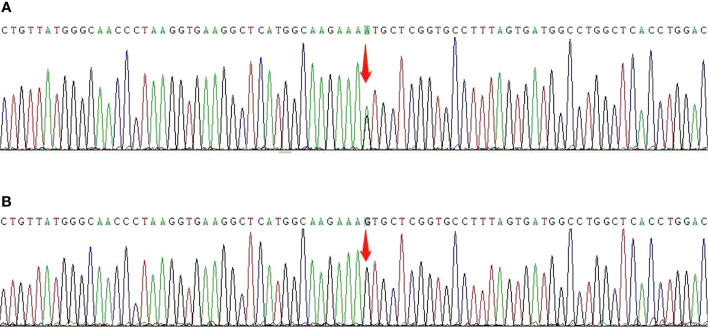
*β-globin* gene sequencing showing the point mutation at c.202G>A before HSCT **(A)** and a normal sequence after HSCT **(B)**. The arrow shows the position of the mutation.

A 3-month-old boy presented with dark urine, pallor, and mild jaundice. Moderate anemia (Hb 85 g/L) with reticulocytosis (13.4%) was detected in the peripheral blood cell count. Biochemical analysis revealed elevated serum lactate dehydrogenase and hyperbilirubinemia. Coombs and autoantibody test results were negative. DNA test results for thalassemia were normal. The patient was diagnosed with hemolytic anemia. The Hb level decreased to 58 g/L when he was 10 months. Physical examination revealed generalized icterus accompanied by marked splenomegaly (3 cm below the costal margin). From then on, he was treated conservatively with multiple blood transfusions of an average of 2.5 units every month to maintain a Hb level of 90 g/L. He was re-evaluated at 2 years of age. Malnutrition or growth retardation was not observed. A blood smear examination revealed erythrocyte abnormalities with marked anisocytosis, poikilocytosis, and basophilic stippling. Specific enzyme activity assays for glucose-6-phosphate dehydrogenase, phosphate isomerase, pyruvate kinase, and pyrimidine 5’-nucleotidase showed normal findings. Hb electrophoresis revealed elevated fetal Hb (21%) without anomalous Hb. Both heat stability and isopropanol tests showed positive results. DNA sequencing of the globin genes were subsequently performed and identified a heterozygous mutation in Hbβ codon 67, variant c.202G>A Val-Met (**Figure 1A**, GenBank accession No. OQ718455). The boy was definitively diagnosed with unstable hemoglobinopathy and Hb Bristol-Alesha. He had no family history of hemolytic anemia, and his parents were found to be normal based on DNA sequencing of globin chains. Deferasirox was administered at 4 years of age because of iron overload, with a serum ferritin level >2000 ng/mL (**Supplementary Figure 1**). However, the serum ferritin level did not decrease below 1000 ng/mL on periodical tests. Moreover, conventional hepatic magnetic resonance imaging at 6 years of age revealed a diffuse abnormal signal, indicating iron deposition in the liver.

**Figure 2:**
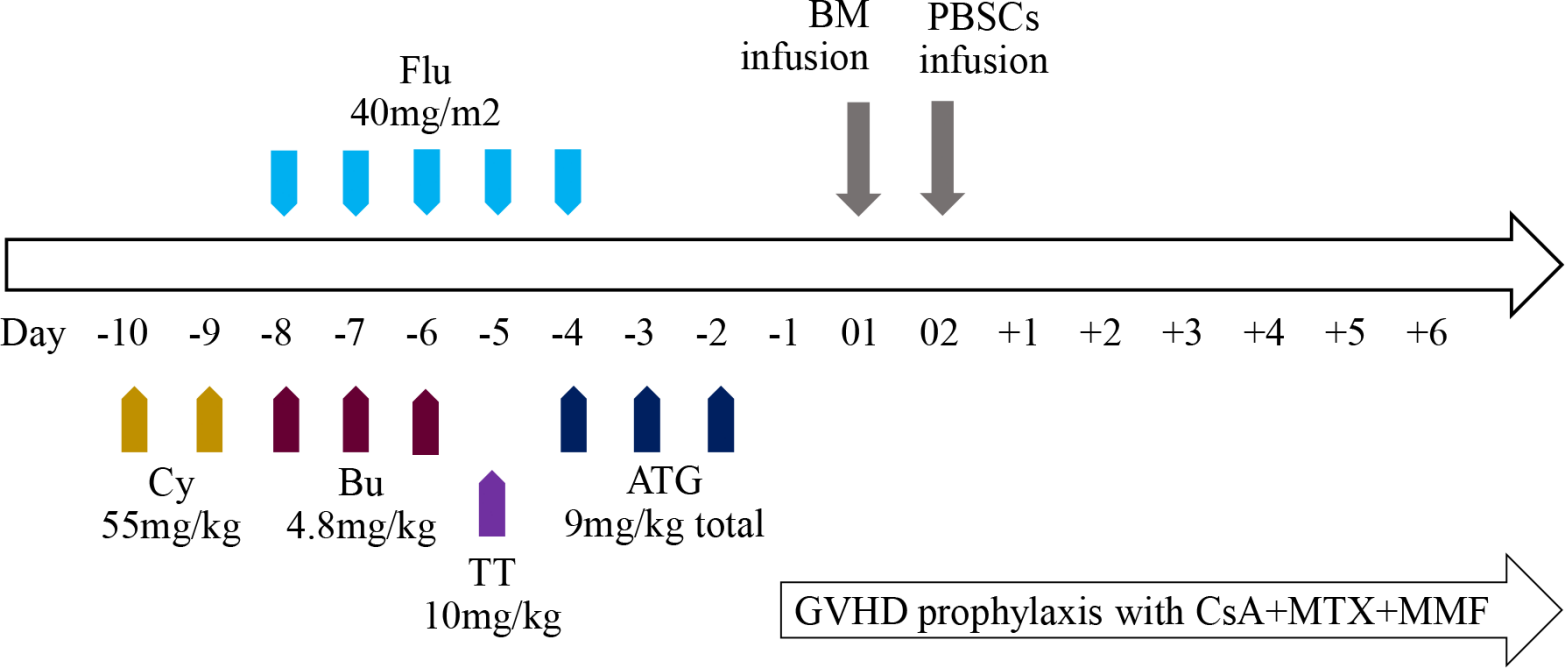
Overview of the haplo-HSCT protocol.

In view of serious transfusion dependence and the possibility of end-organ injury due to iron overload, HSCT was performed at the age of 6 years. As the patient did not have matched siblings or unrelated donors, he underwent haplo-HSCT with a myeloablative conditioning regimen (**Figure 2**). The donor was his father who has a human leukocyte antigen (HLA) 5/10 allele match. Donor-specific anti-HLA antibodies (DSAs) of the recipient before HSCT were negative; therefore, desensitization strategies were not conducted. The conditioning regimen consisted of cyclophosphamide (Cy) 55 mg/kg per day from days −10 to −9, busulfan (Bu) 4.8 mg/kg per day from days −8 to −6, fludarabine (Flu) 40 mg/m^2^ per day from days −8 to −4, and thiotepa (TT) 10 mg/kg on day −5. Anti-thymoglobulin (ATG) was also added to the regimen at a total dose of 9 mg/kg from days −4 to −2. Graft-versus-host disease (GVHD) prophylaxis included cyclosporine A (CsA), mycophenolate mofetil (MMF), and methotrexate (MTX). CsA was intravenously injected from day −1 to +25 and then orally administered to maintain its blood concentration at 200–250 ng/mL, and MMF was administered from day +1 to day +30 at a dose of 20–30 mg/kg per day. Short-term MTX was administered on days +1, +3, +6, and +11 at 15 mg/m^2^, 10 mg/m^2^, 10 mg/m^2^, and 10 mg/m^2^, respectively. Considering the different characteristics of hematopoietic stem cells from the bone marrow (BM) and peripheral blood stem cells (PBSCs), the patient received an infusion of a combination of BM and PBSCs mobilized by granulocyte colony-stimulating factor (G-CSF) on days 01 and 02, respectively, to improve engraftment and reduce the occurrence of GVHD. The cell dose of infusion was 21.44×10^8^/kg of nucleated cells, 7.64×10^6^/kg of CD34^+^ cells, and 2.14×10^6^/kg of CD3^+^ cells. The time to neutrophil and platelet engraftment was 15 and 17 days, respectively. The patient developed cytomegalovirus reactivation and BKV^+^ hemorrhagic cystitis are on days 24 and 32, respectively, which were controlled using antiviral drugs and intravenous immunoglobulin. Grade II acute GVHD (skin type) was also detected on day 25 post-transplantation and was controlled by the infusion of CD52 monoclonal antibody. The patient showed complete chimerism on day 30, which was sustained at 3, 6, 9, 12, 18, and 24 months post-HSCT. Furthermore, the former mutation of Hbβ was not detected using DNA resequencing (**Figure 1B**, GenBank accession No. OQ718456). During a period of 2 years follow-up, Hb was maintained at a high level of >110 g/L. The serum ferritin level gradually decreased to 1100ng/mL and no chronic GVHD was observed. To date, he has been transfusion-free.

## 3 Discussion and conclusions

Unstable hemoglobinopathies are a group of rare congenital diseases that present with nonimmune hemolytic anemia of varying degrees. To date, more than 100 unstable Hb variants have been discovered using DNA sequencing (9). The incidence of this uncommon disorder is relatively low; therefore, only individual cases of each variant have been reported. Hb Bristol-Alesha is caused by a mutation of the β-globin gene, leading to the replacement of Val by Met. The Met residue is subsequently modified to Asp, probably *via* oxidative mechanisms (10). To the best of our knowledge, only 15 cases of this variant have been reported, all of which were dependent on blood transfusion (**Table 1**) (8, 11–23). According to this case and a previous analysis conducted on Hb Bristol-Alesha, it is always caused by *de novo* mutations. In addition, these cases have been reported in subjects of different origins, suggesting that this variant does not have a regional or racial propensity.

**Table 1:** Clinical features of published cases of Hb Bristol-Alesha.

Reference	Nationality	Age at onset (month)	Sex	Hb (g/L)	Family history	Transfusion required	Splenectomy	Transplantation	Growth retardation	Specific features
Steadman et al., 1970 (11)	British	16	M	70	No	Yes	Yes	No	No	Hemolytic crisis, subarachnoid hemorrhages, and rheumatic fever
Ohba et al., 1985 (12)	Japanese	7	M	70-80	NR	1–1.5 units every 2 months	Yes	No	No	Gall stones
		NR	M	50-60	NR	4 units every month	No	No	Yes	Gall stones and hepatitis C
Aseeva et al., 1992 (13)	Russian	NR	M	70-80	NR	Yes	Yes	No	Yes	Thalassemic facies
Molchanova et al., 1993 (8)	Russian	2	M	80-90	No	6–8 times every year	Yes	No	Yes	Bone changes
Kano et al., 2004 (14)	Japanese	6	M	50-60	No	2 units every month	Yes	No	Yes	Hemolytic crisis and aplastic crisis
Brockmann et al., 2005 (15)	Germany	4	F	50-60	No	Once a month	Partial	No	NR	Central cyanosis, arterial hypertension, and Moyamoya syndrome
Eberle et al., 2007 (16)	Argentinean	6	F	84	No	Yes	Yes	No	Yes	Peculiar facial appearance
Jiang et al., 2016 (17)	Chinese	3	F	56	No	Yes	No	No	Yes	None
Pedroso et al., 2017 (18)	Brazilian	6	F	46	No	Once a month	No	Yes, but failed because of rejection	NR	Bone changes
Hamid et al., 2019 (19)	Iranian	NR	M	65	No	Yes	No	No	NR	None
Su et al., 2019 (20)	Chinese	4	M	61	No	Once every 2 weeks	No	No	No	None
Rizzuto et al., 2021 (21)	Spanish	4	M	70-80	No	8 units every year	Yes	No	Yes	None
Li et al., 2022 (22)	Chinese	3	F	60	No	Yes	No	Yes	Yes	None
Corrons et al., 2022 (23)	Indian	4	F	60	NR	Once a month	Yes	No	No	None

NR, not reported.

Our patient presented with a very unstable Hb phenotype, and abnormal Hb levels could not be detected on electrophoresis. Therefore, DNA sequencing of the globin genes was necessary for an accurate diagnosis. There is evidence that hyperunstable Hb variants cause symptoms clinically similar to those associated with TM (14). This patient developed severe hemolytic anemia and marked splenomegaly, requiring frequent transfusions and iron chelation to improve anemia and suppress ineffective erythropoiesis. It should be noted that transfusions are not risk-free. On the one hand, repetitive RBC transfusions expose patients to a high risk of developing alloantibodies, which can limit the availability of compatible RBCs for future transfusions and increase the risk of transfusion complications (24, 25). On the other hand, progressive iron loading resulting from repeated RBC transfusions and increased absorption of dietary iron is most likely to induce tissue-specific siderosis in the liver, myocardium, pancreas, and pituitary gland (26). As a result, patients receiving long-term transfusions tend to have organ dysfunction, which worsens with increasing age (27). Furthermore, the high economic burden related to lifelong transfusion therapy and iron chelation, together with the high usage of health care resources, provides more insights into possible curative therapies (28). Although splenectomy in later years seems to modestly improve the clinical condition in a few patients, it is not a curable option and may increase the risk of infections and thrombotic complications (29).

Currently, allogeneic HSCT offers an available curative approach for hemoglobinopathies. HSCT outcomes from matched identical donors have gradually improved over the past few decades as a result of optimized treatment protocols, leading to an overall survival (OS) rate exceeding 90% for TM and SCD (30–32). A retrospective follow-up study evaluated the long-term outcomes in 137 patients with TM who underwent allogeneic HSCT. The 39-year OS and disease-free survival rates were 81.4% and 74.5%, respectively, indicating a definitive cure for the majority of patients (33). For those lacking HLA-matched sibling donors or unrelated donors, HLA-haploidentical donors (in pediatrics, in most cases a parent) are increasingly considered and used (34). However, owing to the relatively low prevalence, there are currently no practical recommendations or treatment guidelines for patients with unstable hemoglobinopathies. Although allogeneic HSCT is the only curative option for severe cases, experience with this procedure is limited. To date, successful cases of HSCT have rarely been reported (21, 22, 35–38). These cases are summarized in **Table 2**.

**Table 2:** Summary of allogeneic HSCT for unstable hemoglobinopathies.

Reference	Age at SCT	Hb variant name Genotype	Donor type	Hb level before SCT (g/L)	Preparative regimen	Manipulation of grafts	CD34^+^ infusion dose (cells/kg)	Engraftment of neutrophil	Engraftment of platelet	Transplantation-related complications	Outcomes
Urban et al. (35),	18m	Hb Olmsted HBB:c.425T>G	MSD	55	Bu,/Cy/ATG	Unmanipulated	15.2×10^6^	+14d	+21d	None	100% donor chimerism on +23d, disappearance of hepatosplenomegaly, and normal hemoglobin level
Croteau et al. (36),	3y	Hb Boston-Kuwait HBB:c.421insT	MSD	74	Bu/Cy/ATG	Unmanipulated	NR	+28d	+25d	NR	>97% donor chimerism on +60d and transfusion-independent
Kumar et al. (37),	5y	Hb Hammersmith HBB:c.128T>C	Haplo	NR	Flu/Bu/Cy/ TBI/RTX	TCR αβ/CD19-depleted	20.5×10^6^	+12d	NR	Grade 1 aGVHD CMV reactivation	100% donor chimerism on +30d and symptom-free
Li et al. (22),	15m	Hb Bristol-Alesha HBB:c.202G>A	Haplo+ Cord	60	Flu/Bu/Cy	Unmanipulated	6.8×10^6^	+11d	+18d	None	95% donor chimerism on +30d and normal Hb level
Chan et al. (38),	9y	Hb Hammersmith HBB:c.128T>C	Haplo	NR	Flu/Cy/TT/ Treo/TLI	TCR αβ/CD45RA-depleted	110×10^6^	+19d*	+36d	EBV viremia Adenoviremia Grade 2 skin and gut aGVHD	92% donor chimerism on +14d of the second SCT, durable engraftment by repeated DLIs, and transfusion-independent
Rizzuto et al. (21),	4y	Hb Zunyi HBB:c.442T>C	NR	82	–	–	–	–	–	–	Successful**
	3y	Hb Mokum HBB:c.442T>A	NR	79	–	–	–	–	–	–	Successful**

*The patient underwent two transplantations. The first transplantation resulted in acute graft rejection. Neutrophils engrafted on day 19 at the second transplantation.

** The details of HSCT were not documented.

(aGVHD, acute graft-versus-host disease; ATG, anti-thymocyte globulin; Bu, busulfan; Cy, cyclophosphamide; DLI, donor leukocyte infusion; Flu, fludarabine; HSCT, hematopoietic stem cell transplantation; MSD, matched sibling donor; NR, not reported; RTX, rituximab; SCT, stem cell transplantation; TBI, total body irradiation; Treo, treosulfan; TLI, total lymphoid irradiation; TT, thiotepa).

Considering the severe hemolytic phenotype of our patient and the unavailability of matched donors, haplo-HSCT was planned. Similar to TM and SCD, disease-specific features, including hyperplastic BM and allosensitization due to multiple transfusions, render haplo-HSCT a high risk for graft failure (GF) (39, 40), and this may be the main obstacle. Patients receiving transfusions have a higher prevalence of anti-HLA antibodies, which are defined as DSAs when their specificity corresponds to a mismatched donor antigen (41). DSAs are a major cause of primary GF, including graft rejection (GR) and poor graft function (PGF) in patients receiving haplo-HSCT (42, 43). The rejection rate is much higher in the DSA-positive group than in the DSA-negative group, and DSA-positive patients have worse OS and inferior progression-free survival (44, 45). Therefore, anti-HLA antibodies should be evaluated in all haplo-HSCT recipients, especially in those receiving multiple transfusions (46). Desensitization could be applied in recipients with pretransplant DSAs if alternative donor is unavailable (47). We performed DSA detection in our patient before haplo-HSCT and obtained a negative result. Consequently, neither GR nor PGF was found in the subsequent transplantation process.

The conditioning regimen also plays a vital role in stem cell engraftment, and the results from thalassemia could provide a valuable reference. Myeloablative preconditioning regimens with more advanced immunosuppression concepts and application of T-cell depletion strategies, either ex vivo T-cell depletion (CD3^+^/CD19^+^ or TCRαβ^+^/CD19^+^ depletion) or *in vivo* T-cell depletion (post-transplant Cy or G-CSF primed peripheral blood graft with ATG), have significantly improved the outcomes of haplo-HSCT (48–52). A previous study that adopted a novel NF-08-TM conditioning regimen reported high long-term OS and thalassemia-free survival rates in China (53, 54). More recently, additional research comparing HLA fully matched and mismatched HSCT for patients with TM demonstrated similar survival outcomes and incidences of complications (except for acute GVHD) based on the modified NF-08-TM regimen, adjusting the ATG dose according to HLA compatibility (55). Similar to that used in patients with thalassemia, a myeloablative regimen including Bu, Cy, Flu, TT, and ATG (at a total dose of 9 mg/kg) was administered in our patient. The boy achieved full donor chimerism and has remained transfusion-free to date. Notably, he developed CMV and BKV infection after transplantation. It has been speculated that viral infection complications are associated with delayed immune reconstitution caused by the addition of ATG (56–58).

In conclusion, unstable hemoglobinopathy is a rare disease, and a subset of patients present with severe hemolytic anemia. Based on our experience from this case, haplo-HSCT may be a curative option for patients with a transfusion-dependent phenotype of unstable hemoglobinopathy when matched donors are not available. Further clinical studies are required before haplo-HSCT can be widely applied in clinical practice.

## Data availability statement

The datasets presented in this study can be found in online repositories. The names of the repository/repositories and accession number(s) can be found below: OQ718455, OQ718456 (NCBI GenBank database).

## Ethics statement

The studies involving human participants were reviewed and approved by the Sixth Medical Center of PLA General Hospital, China. Written informed consent to participate in this study was provided by the participants’ legal guardian/next of kin. Written informed consent was obtained from the participant/patient(s) for the publication of this case report.

## Author contributions

QZ and YH were responsible for data curation and drafting of the manuscript. QS, NL, HS, MW, and JX contributed to patient care and follow-up. HY collected the data. XT conceived and designed the study and guided the article revision. All the authors contributed to the manuscript and approved the submitted version.
