# Supplementary material for: Case report: Curing a rare, unstable hemoglobin variant Hb Bristol-Alesha using haploidentical hematopoietic stem cell transplantation
Source: Front Immunol. 2023 Jun 30;14:1188058. doi: 10.3389/fimmu.2023.1188058 (PMC10348747; doi:10.3389/fimmu.2023.1188058)
Supplement: Supplementary file 1 [file DataSheet_1.pdf]

Supplementary Material

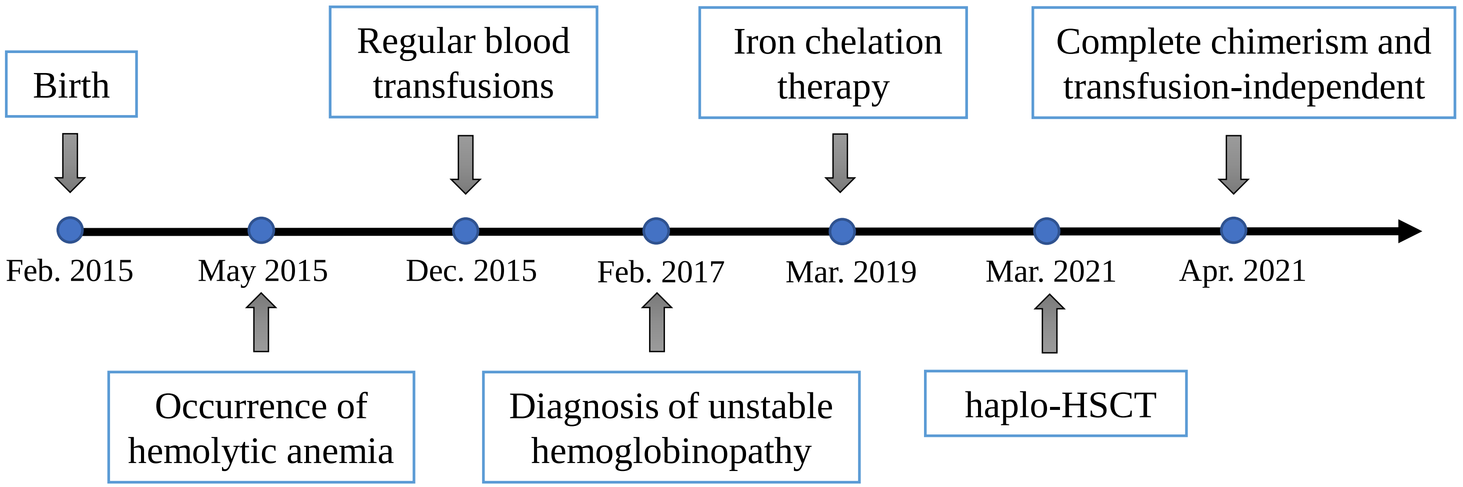

Supplementary Figure 1. Timeline depicting the disease course and interventions of the patient. The timeline illustrates the different events in the course of the patient's treatment and disease progression.
